# Childhood intelligence attenuates the association between biological ageing and health outcomes in later life

**DOI:** 10.1038/s41398-019-0657-5

**Published:** 2019-11-28

**Authors:** Anna J. Stevenson, Daniel L. McCartney, Robert F. Hillary, Paul Redmond, Adele M. Taylor, Qian Zhang, Allan F. McRae, Tara L. Spires-Jones, Andrew M. McIntosh, Ian J. Deary, Riccardo E. Marioni

**Affiliations:** 1 Centre for Genomic and Experimental Medicine, Institute of Genetics and Molecular Medicine, University of Edinburgh, Edinburgh, UK; 2 Department for Psychology, University of Edinburgh, Edinburgh, UK; 3 Institute for Molecular Bioscience, University of Queensland, Brisbane, QLD 4072 Australia; 4 UK Dementia Research Institute, Edinburgh Medical School, University of Edinburgh, Edinburgh, UK; 5 Centre for Discovery Brain Sciences, University of Edinburgh, Edinburgh, UK; 6 Division of Psychiatry, Centre for Clinical Brain Sciences, University of Edinburgh, Edinburgh, UK; 7 Centre for Cognitive Ageing and Cognitive Epidemiology, University of Edinburgh, Edinburgh, UK

**Keywords:** Genetics, Genomics

## Abstract

The identification of biomarkers that discriminate individual ageing trajectories is a principal target of ageing research. Some of the most promising predictors of biological ageing have been developed using DNA methylation. One recent candidate, which tracks age-related phenotypes in addition to chronological age, is ‘DNAm PhenoAge’. Here, we performed a phenome-wide association analysis of this biomarker in a cohort of older adults to assess its relationship with a comprehensive set of both historical, and contemporaneously-measured, phenotypes. Higher than expected DNAm PhenoAge compared with chronological age, known as epigenetic age acceleration, was found to associate with a number of blood, cognitive, physical fitness and lifestyle variables, and with mortality. Notably, DNAm PhenoAge, assessed at age 70, was associated with cognitive ability at age 11, and with educational attainment. Adjusting for age 11 cognitive ability attenuated the majority of the cross-sectional later-life associations between DNAm PhenoAge and health outcomes. These results highlight the importance of early life factors on healthy older ageing.

## Introduction

A key objective in ageing research is the development of biomarkers that distinguish individuals on different ageing trajectories. Owing to the distinct and calculable pattern of age-related changes in DNA methylation across the genome with chronological age, a number of DNA methylation-based biomarkers of ageing, or ‘epigenetic clocks’, have been developed. Accelerated epigenetic ageing has been linked with a number of age-related morbidities and with increased risk of mortality^1,2^. Whereas the first-generation of epigenetic clocks were developed using solely chronological age as the reference, a more-recent effort additionally incorporated age-related phenotypes including blood cell profiles and inflammatory markers^3^. This newer clock, termed DNAm PhenoAge, aimed to capture a truer and more efficacious epigenetic biomarker of physiological age, one which discriminates morbidity and mortality more definitively among individuals of the same chronological age.

DNAm PhenoAge was found to associate with diverse morbidities and mortality, with improved predictive power over other epigenetic clocks^3^. However, many of the associations were with general composite indices of health outcomes, rather than individual phenotypes. Moreover, the associations between DNAm PhenoAge and early life factors are currently unknown. It has been acknowledged that childhood and life-course traits and circumstances might have an enduring impact on later health. For example, greater childhood deprivation, lower childhood intelligence, relatively little formal education, and more manual adult occupations have been associated with increased morbidity and mortality risk in older age^4–7^. Accordingly, for a more complete picture of the validity of DNAm PhenoAge, in addition to testing its relationship with individual ageing outcomes and mortality, it would be desirable to examine whether it can be predicted by these life history variables.

Here, we conduct a phenome-wide association study (PheWAS), in which multiple phenotypes are related to a single outcome, to investigate the link between accelerated DNAm PhenoAge and a comprehensive set of both historical, and contemporaneously-assessed, phenotypes in a large, longitudinal cohort study of ageing: the Lothian Birth Cohort 1936 (LBC1936). This cohort is unusually valuable because data are available on their general cognitive ability and social circumstances at age 11, which can inform the understanding of possible early life confounders of later-life outcomes.

## Methods

### Study population

The Lothian Birth Cohort 1936 (LBC1936) is a longitudinal study of ageing. The cohort comprises a community-dwelling sample of participants born in 1936, most of whom undertook a general intelligence test—the Moray House Test No. 12—in 1947, aged ~11 years. In total 1091 participants were recruited to the study at a mean age of ~70 years (Wave 1), and have subsequently been re-examined at three furthers waves, aged ~73, 76 and 79 years. Participants have been comprehensively phenotyped at each wave of the study with data collected on cognitive measures, physical and health outcomes, genetics, lifestyle factors and psycho-social aspects of ageing. Full details on the background, recruitment and data collection procedures of the study are provided elsewhere^8,9^.

### Ethics and consent

Ethical permission for LBC1936 was obtained from the Multi-Centre Research Ethics Committee for Scotland (MREC/01/0/56) and the Lothian Research Ethics Committee (Wave 1: LREC/2003/2/29) and the Scotland A Research Ethics Committee (Waves 2, 3 and 4: 07/MRE00/58). Written informed consent was obtained from all participants.

### LBC1936 DNA methylation

The LBC1936 methylation profiling has been fully detailed previously^10,11^. In brief, DNA was extracted from whole-blood samples at Wave 1 (baseline—age ~70 years) of the study and methylation was measured at 485,512 probes. Quality control analysis resulted in the removal of CpG sites with a low detection rate (<95% at *p* < 0.01). Probes with low quality (inadequate hybridisation, bisulfite conversion, nucleotide extension, and staining signal) were additionally identified and removed after manual inspection of the array control probe signals. Finally, probes with a low call rate (<450,000 probes detected at *p* < 0.01), XY probes and samples in which the predicted sex did not match the reported sex, were excluded.

### DNAm PhenoAge

The DNAm PhenoAge biomarker was developed in a two-step process by Levine et al.^3^. In brief, a novel measure of ‘phenotypic age’ was developed using penalised regression where the hazard of ageing-related mortality was regressed on 42 clinical markers from the third National Health and Nutrition Examination Survey (NHANES-III; *n* = 9926, age: >20 years). The optimal model selected nine variables (albumin, creatinine, serum glucose (HbA_1c_), C-reactive protein (CRP), percentage lymphocytes, mean cell volume, red cell distribution width, alkaline phosphatase, and white blood cell count) in addition to chronological age for inclusion in the phenotypic age predictor. This predictor was derived independently of DNA methylation data. Phenotypic age was then calculated in an independent data set—the Invecchiare in Chianti (InCHIANTI) cohort (*n* = 456 at two time-points, age range: 21–100 years)^12^. Finally, a penalised regression model of DNA methylation on phenotypic age in InCHIANTI generated a DNA methylation proxy of phenotypic age (labelled DNAm PhenoAge) based on methylation profiles at 513 CpGs. DNAm PhenoAge allows for an estimate of phenotypic age from a single array, obviating the need for multiple assays to measure the nine blood-based components of phenotypic age.

DNAm PhenoAge was calculated in LBC1936 by multiplying CpG methylation levels with the regression weights from the above analysis^3^. One CpG (cg06533629) from the 513 used in the original computation was not available in the LBC1936 methylation data. At Wave 1, 889 individuals (81.5%) within the LBC1936 cohort had full methylation data available for the calculation of DNAm PhenoAge.

### Phenotypic data

The PheWAS included 107 phenotypes broadly associated with health and wellbeing. The phenotypes encompassed seven subgroups: blood, cardiovascular, cognitive, personality and mood, lifestyle, physical, and life history, and were measured on a binary (*n* = 15), continuous (*n* = 89), or ordinal (*n* = 3) scale. Six of the phenotypes included in the PheWAS (white cell counts, blood glucose, CRP, creatinine, albumin and mean cell volume) were incorporated in the original phenotypic age estimate.

Descriptive statistics for the phenotypes are presented in Supplementary Table 1. Data collection protocols are detailed in Supplementary File 1 and have been described fully previously^13^.

### Statistical analysis

DNAm PhenoAge acceleration (DNAm PhenoAgeAccel)—defined as the residuals resulting from regressing DNAm PhenoAge on chronological age—was calculated for all participants at LBC1936’s Wave 1, at a mean age of 70 years. DNAm PhenoAgeAccel captures the difference between DNAm PhenoAge and chronological age, with positive values indicating a faster epigenetic ageing rate. The acceleration measure was used in analyses in order to account for the correlation between DNAm PhenoAge and chronological age.

Linear regression models were used to obtain the associations between the continuous variables with DNAm PhenoAgeAccel. All continuous variables were scaled to have a mean of zero and unit variance to ensure comparable effect sizes across all traits. Generalised linear models with a logit link function (logistic regression) were used to investigate the association between the binary variables and DNAm PhenoAgeAccel, and ordinal regression models were used for the ordered categorical measures of smoking (three levels), physical activity (five levels) and occupational social class (six levels). DNAm PhenoAgeAccel was the independent variable of interest in each regression model. Height and smoking status (Wave 1) were included as covariates in the models for lung function (forced expiratory volume FEV_1_; forced vital capacity: FVC; forced expiratory ratio: FER; and peak expiratory flow: PEF). All models were adjusted for chronological age and sex. To investigate the influence of childhood cognitive ability, all models that showed significant associations with DNAm PhenoAgeAccel were repeated adjusting for age 11 IQ scores.

In the longitudinal analysis, linear mixed-effects models were used to assess if baseline DNAm PhenoAgeAccel was associated with longitudinal change over the four waves of data (~70 years to ~79 years) in a subset of the cognitive and physical phenotypes that are known to decline with age and correlate with functional impairment. Here, Wave 1 DNAm PhenoAgeAccel was included as a fixed-effect interaction with chronological age, and participant was added as a random-effect intercept term. As above, height and smoking status were included in the models for lung function, and all models co-varied for sex. Cox proportional-hazard models were implemented for survival (time-to-death) analyses.

Given the correlation structure between phenotypes within each group, we applied correction for multiple testing using the false discovery rate (FDR) method to each group of variables individually^14^. We additionally tested how results changed using a more conservative Bonferroni adjustment; first, a principal component analysis was run on the 107 phenotypes, which indicated 80% of the variance was explained by 47 principal components. A Bonferroni correction of 0.05/47 (adjusted *P* < 0.001) was then applied.

Statistical analysis was conducted in R version 3.5.0 using the ‘lm’ and ‘glm’ function in the ‘stats’ library and the ‘lme4’, ‘lmerTest’, ‘rms’ and ‘Survival’ packages^15–20^.

## Results

### Cohort information

Details of the baseline (Wave 1) characteristics of LBC1936 are presented in Supplementary Table 1. In all, 49.5% of the cohort was female. Mean chronological age for both males and females was 69.5 years (SD 0.8) and mean DNAm PhenoAge was 57.8 years (females = 56.7 (SD = 8.1), males = 58.8 (SD 8.2)). The discrepancy between the chronological and epigenetic age measures is probably reflective of the overall good health of the cohort.

### PheWAS

Only associations with an FDR-corrected significant *p* value (<0.05) are presented here and in Fig. 1. *P* values represent the significance of the association between DNAm PhenoAgeAccel and individual phenotypes. Full results are presented in Supplementary Figs 1–7 and Supplementary Table 2.

**Fig. 1:**
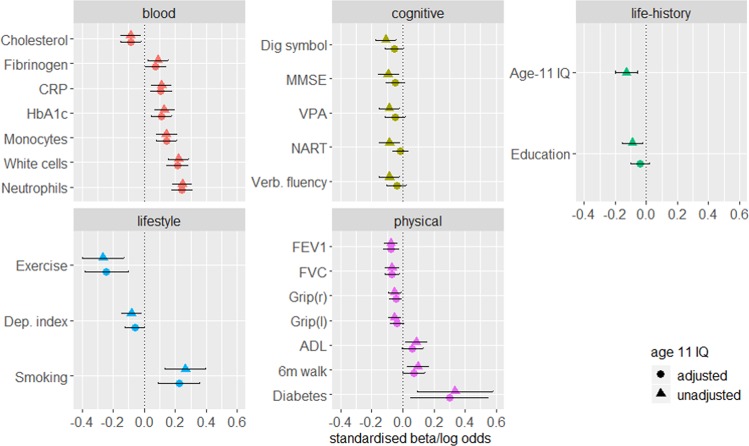
FDR-corrected significant associations between DNAm PhenoAgeAccel and blood, cognitive, lifestyle, physical and life-history variables. Standardised model *β* coefficients (for continuous variables) or log odds (for binary variables) are presented along the *x* axes. Phenotypes are presented along the *y* axes. Error bars show the 95% confidence interval. CRP: C-reactive protein; Dep: deprivation; VPA: verbal paired associates; verb: verbal; NART: National Adult Reading Test; MMSE: Mini-Mental State Examination; grip: grip strength; FEV1: forced expiratory volume in 1 s; FVC: forced vital capacity; ADL: activities of daily living (Townsend Disability Scale).

### Blood

Significant associations between DNAm PhenoAgeAccel and blood phenotypes are presented in Fig. 1. Full results are presented in Supplementary Fig. 1 and Supplementary Table 2. Of the six measures included in the phenotypic age reference, three significant positive associations were found with DNAm PhenoAgeAccel: white cell counts (*β* = 0.22, SE = 0.03, *p* = 2.5 × 10^−9^) HbA_1c_ (*β* = 0.13, SE = 0.03, *p* = 5.8 × 10^−4^) and C-reactive protein (CRP; *β* = 0.11, SE = 0.03, *p* = 0.006).

Significant positive relationships were additionally identified between DNAm PhenoAgeAccel and neutrophils (*β* = 0.25, SE = 0.03, *p* = 2.9 × 10^−12^), monocytes (*β* = 0.14, SE = 0.03, *p* = 1.6 × 10^−4^) and fibrinogen (*β* = 0.09, SE = 0.03, *p* = 0.043). A negative association was found between DNAm PhenoAgeAccel and total cholesterol levels (*β* = −0.09, SE = 0.03, *p* = 0.043).

### Cardiovascular

No significant associations were found between DNAm PhenoAgeAccel and any of the cardiovascular variables (Supplementary Fig. 2 and Supplementary Table 2, FDR- corrected *p* ≥ 0.12).

### Cognitive

Significant associations between DNAm PhenoAgeAccel and cognitive phenotypes are presented in Fig. 1. Full results are presented in Supplementary Fig. 3 and Supplementary Table 2. Higher DNAm PhenoAgeAccel associated with lower scores on one test of the processing speed domain (digit symbol coding: *β* = −0.11, SE = 0.03, *p* = 0.015), one test of the memory domain (verbal paired associates: *β* = −0.09, SE = 0.03, *p* = 0.034), the mini-mental state examination (*β* = −0.10, SE = 0.03, *p* = 0.034) and two tests of crystallised ability (national adult reading test: *β* = −0.09, SE = 0.03, *p* = 0.034; verbal fluency: *β* = −0.09, SE = 0.03, *p* = 0.034).

### Personality and mood

No significant associations were found between DNAm PhenoAgeAccel and any of the personality and mood phenotypes (Supplementary Fig. 4 and Supplementary Table 2).

### Physical

Significant associations between DNAm PhenoAgeAccel and physical phenotypes are presented in Fig. 1. Full results are presented in Supplementary Fig. 5 and Supplementary Table 2. We found significant inverse associations between DNAm PhenoAgeAccel and FEV_1_ (*β* = −0.07, SE = 0.02, *p* = 0.023), FVC (*β* = −0.07, SE = 0.02, *p* = 0.023), and grip strength in both right and left hands (both: *β* = −0.05, SE = 0.02, *p* = 0.045). Higher DNAm PhenoAgeAccel was associated with a self-reported diagnosis of diabetes (OR = 1.39, 95% CI (1.01, 1.78), *p* = 0.038), a slower six metre walk time (*β* = 0.01, SE = 0.03, *p* = 0.038), and a higher score on the Townsend’s Disability Scale (activities of daily living; *β* = 0.09, SE = 0.03, *p* = 0.045).

### Lifestyle

Significant associations between DNAm PhenoAgeAccel and lifestyle phenotypes are presented in Fig. 1. Full results are presented in Supplementary Fig. 6 and Supplementary Table 2. A significant inverse association was found between baseline DNAm PhenoAgeAccel and the deprivation index, such that a higher epigenetic age associated with a score indicative of greater deprivation (*β* = −0.08, SE = 0.03, *p* = 0.025). In addition, a higher DNAm PhenoAgeAccel was associated with lower levels of physical activity (OR = 0.77, 95% CI (0.67, 0.88), *p* = 0.0003) and with higher odds of being either a current, or an ex-, smoker, compared with a never smoker (OR = 1.31, 95% CI (1.15, 1.49), *p* = 0.0003).

### Life-history

Significant associations between DNAm PhenoAgeAccel and life history phenotypes are presented in Fig. 1. Full results are presented in Supplementary Fig. 7 and Supplementary Table 2. A higher baseline DNAm PhenoAgeAccel was associated with a lower age 11 IQ (*β* = −0.13, SE = 0.03, *p* = 0.001) and fewer years of education (*β* = −0.09, SE = 0.03, *p* = 0.013).

### Sensitivity analysis

A Bonferroni correction (adjusted *P* < 0.001) resulted in the attenuation of 15 of the 24 originally significant associations identified with DNAm PhenoAgeAccel. Age 11 IQ, smoking and physical activity remained significant, in addition to the cognitive test of processing speed (digit symbol coding) and five of the blood variables. These results are presented in Supplementary Table 2.

### DNAm PhenoAge and survival

**Fig. 2:**
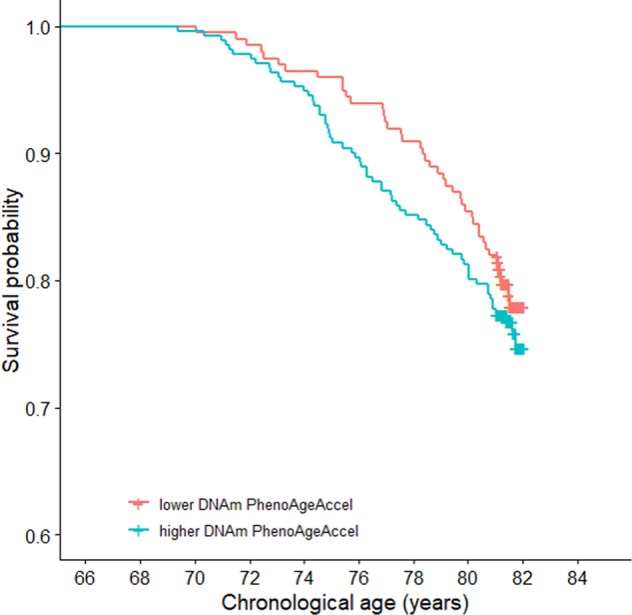
**Survival probability by quartiles of DNAm PhenoAgeAccel adjusted for sex and chronological age.**

We tested the association of DNAm PhenoAge with all-cause mortality (*n*_deaths_ = 209, over 9 years of follow-up, average age of death = 76.8 years, SD 3.3) and found that a higher DNAm PhenoAgeAccel was significantly associated with risk of death (HR = 1.17 per SD increase in DNAm PhenoAgeAccel, 95% CI (1.02, 1.34), *p* = 0.025). A Kaplan–Meier survival curve for DNAm PhenoAgeAccel, split into highest and lowest quartiles, is presented in Fig. 2, illustrating the higher mortality risk for those with a higher DNAm PhenoAgeAccel.

To assess the performance of DNAm PhenoAgeAccel in relation to mortality risk, we ran additional survival analyses using other markers previously associated with mortality^21–24^. Each of the assessed markers outperformed DNAm PhenoAgeAccel: BMI (HR = 1.21 per SD increase in BMI, 95% CI (1.07, 1.38), *p* = 0.003), smoking status (HR = 1.78 for ex-smokers, 95% CI (1.29, 2.45), *p* = 0.0004; HR = 4.38 for current smokers, 95% CI (3.01, 6.36), *p* = 9.23 × 10^−15^), grip strength (HR = 0.63 per SD increase in mean grip strength, 95% CI (0.51, 0.78), *p* = 1.59 × 10^−5^) and walking speed (HR = 1.39 per SD increase in walking speed, 95% CI (1.27, 1.52), *p* = 1.74 × 10^−12^). Kaplan–Meier plots for each of these variables are presented in Supplementary Fig. 8.

### Adjusting for Age 11 IQ

To test for potential confounding of the associations by childhood intelligence, the models for all of the significant associations identified in the PheWAS were re-run adjusting for age 11 IQ. Results are presented in Fig. 1 and Table 1.

**Table 1:** Results before and after adjusting models for age 11 IQ.

	Before age 11 IQ adjustment			After age 11 IQ adjustment		
Phenotype	Standardised *β*	Standard error	FDR-corrected *p*	Standardised *β*	FDR-corrected *p*	% attenuation
Neutrophils	0.245	0.033	**9.26** **×** **10**^**−12**^	0.241	**3.47** **×** **10**^**−11**^	1.7
White cell count	0.222	0.034	**8.24** **×** **10**^**−9**^	0.214	**4.22** **×** **10**^**−8**^	3.7
Monocytes	0.143	0.033	**5.3** **×** **10**^**−4**^	0.143	**2.72** **×** **10**^**−4**^	0
HbA_1c_	0.129	0.033	**0.002**	0.110	**0.004**	14.5
C-reactive protein	0.112	0.033	**0.012**	0.108	**0.006**	3.2
Forced expiratory volume (1 s)	−0.108	0.023	**0.023**	−0.076	**0.006**	30
Forced vital capacity	−0.089	0.023	**0.023**	−0.069	**0.008**	22.1
Cholesterol	−0.086	0.032	**0.043**	−0.088	**0.021**	−2.6
Fibrinogen	0.089	0.034	**0.043**	0.074	0.062	16.6
Grip strength (*r*)	−0.053	0.022	**0.045**	−0.046	0.062	13.3
6-m walk time (*s*)	0.097	0.035	**0.037**	0.073	0.068	24.9
Deprivation index	−0.082	0.033	**0.025**	−0.061	0.088	25.8
Digit symbol coding	−0.11	0.033	**0.015**	−0.057	0.089	48.1
Grip strength (*l*)	−0.053	0.021	**0.045**	−0.040	0.094	25.3
Activities of daily living	0.086	0.034	**0.045**	0.062	0.098	27.6
Mini-mental state examination	−0.095	0.034	**0.034**	−0.048	0.140	49.2
Verbal paired associates	−0.087	0.034	**0.034**	−0.051	0.142	41.9
Years of education	−0.09	0.033	**0.012**	−0.039	0.216	56.3
Verbal fluency	−0.087	0.033	**0.034**	−0.040	0.216	53.5
National adult reading test	−0.087	0.033	**0.034**	−0.015	0.536	82.4
	**Log odds**					
Smoking category	0.267	0.067	**0.002**	0.224	**0.004**	16.2
Physical activity	−0.266	0.069	**0.002**	−0.242	**0.003**	8.9
Diabetes	0.335	0.124	**0.002**	0.303	**0.039**	9.7

Standardised β are presented for continuous variables and log odds for binary or ordinal phenotypes. FDR-corrected significant results are highlighted in bold

The associations with 12 out of the 23 phenotypes that were originally found to be significant in the PheWAS, became non-significant upon adjustment, inclusive of all the cognitive associations. Of the associations that became non-significant, the effect sizes were attenuated by a mean of 39% (range: 13.4–82.4%). The survival model was also no longer significant following adjustment for age 11 IQ (HR = 1.13, 95% CI (0.98, 1.31), *p* = 0.08).

All of the associations with blood phenotypes, excepting fibrinogen, remained significant following adjustment for age 11 IQ, as did the lung function measures, smoking status, physical activity and diabetes.

### Longitudinal association between DNAm PhenoAgeAccel and phenotypes

All the cognitive and physical fitness measures included in the longitudinal analysis showed changes over time that were consistent with declining health (Supplementary Table 3). The rate of decline ranged from 0.02 SDs per year (digit span backwards) to 0.08 SDs per year (telomere length). Six metre walk time increased by 0.1 SDs per year (all *p* ≤ 2 × 10^−8^).

Baseline DNAm PhenoAgeAccel was not found to associate with subsequent change in any of the assessed phenotypes (FDR-corrected *p* ≥ 0.322, Table 2).

**Table 2:** Longitudinal associations between baseline DNAm PhenoAgeAccel and phenotypes.

Phenotype	Standardised *β*	Standard error	Raw *p*	FDR-corrected *p*
Grip strength (*r*)	0.010	0.008	0.189	0.448
Grip strength (*l*)	0.008	0.008	0.288	0.448
Forced expiratory volume (1s)	0.009	0.008	0.261	0.448
Forced vital capacity	0.011	0.009	0.192	0.448
Forced expiratory ratio	0.021	0.015	0.175	0.448
Peak expiratory flow	0.006	0.011	0.613	0.859
Digit span backwards	<0.001	0.013	0.952	0.952
Symbol search	−0.003	0.013	0.821	0.952
Digit symbol coding	0.019	0.009	**0.046**	0.322
Matrix reasoning	0.004	0.013	0.713	0.908
Letter number sequencing	−0.018	0.013	0.180	0.448
Block design	−0.011	0.011	0.282	0.448
6-m walk time (s)	<0.001	0.014	0.945	0.952
Telomere length	−0.025	0.012	**0.041**	0.322

Significant values are bold

## Discussion

In this study, we performed a comprehensive phenome-wide association study to investigate the associations between 107 phenotypes with a new epigenetic estimate of health—DNAm PhenoAge—in a large cohort of older adults. We identified significant correlations at a mean age of 70 years between accelerated DNAm PhenoAge and a number of blood-based, physical, cognitive, and lifestyle phenotypes, in addition to mortality. Importantly, we found that the life-history variables of general cognitive ability, measured at age 11, and number of years of education, related to DNAm PhenoAge at age 70. Moreover, adjustment for age 11 cognitive ability attenuated the majority of the cross-sectional later-life associations between DNAm PhenoAge and health outcomes.

DNAm PhenoAge was developed referencing a surrogate measure of phenotypic age, instead of solely chronological age, in an attempt to better capture the considerable between-person disparities in susceptibility to disease and death. However, our findings suggest that this novel epigenetic clock may be somewhat qualified in its capacity as a biomarker of physiological ageing. Though we identified associations between DNAm PhenoAge and a number of pertinent ageing outcomes, including measures of age-related physical fitness, it did not appear to robustly capture morbidity and mortality outcomes. Although accelerated DNAm PhenoAge did associate with a higher risk of all-cause mortality, its performance was surpassed by four other biomarkers. In addition, we found no evidence to suggest that DNAm PhenoAgeAccel associates with longitudinal phenotypic change, limiting its potential as a prospective biomarker of ageing.

The association between DNAm PhenoAgeAccel with IQ measured almost 60 years previously is a key finding and is indicative of a lifelong, enduring association between cognition and epigenetic ageing. This bolsters cognitive epidemiology findings indicating that general intelligence in childhood, as measured by psychometric tests, is associated with substantial life-course differences in health and morbidity^25,26^. Various, non-exclusive, mechanisms are thought to govern this association, including better health literacy and disease management, higher socioeconomic standing, and the ‘system integrity’ hypothesis, which postulates that higher scores on cognitive ability tests are capturing a systemic level of good functioning rather than isolated brain efficiency^27^. It is possible that individual differences in DNAm PhenoAgeAccel in older age are, in part, caused by intelligence differences over the life-course, or that both are a result of a shared genetic architecture or early environmental event.

All of the associations found between DNAm PhenoAgeAccel and contemporaneous cognitive, physical fitness, education and socioeconomic status measures ceased to be significant following adjustment for age 11 IQ. The only exceptions were the measures of lung function, diabetes, blood biomarkers (that are highly correlated with the component parts of Phenotypic Age), smoking and physical activity. This indicates that the relationship between DNAm PhenoAgeAccel and the majority of our contemporaneously measured phenotypes may be partially mediated through childhood cognitive ability. These findings are consistent with a theory of reverse causation.

Although we focused on testing for confounding via adjustment for childhood cognitive ability, it is unlikely that is the sole confounding variable. For instance, life-course smoking and socioeconomic status are associated with accelerated DNAm PhenoAge and may similarly confound the identified associations. However, childhood intelligence typically predates these confounders, allowing for the stratification of individuals from a very young age, prior to the manifestation of other traits.

### Strengths and limitations

This is the first independent test of DNAm PhenoAge in a large cohort of older adults with the availability of historical variables, as well as longitudinal measures for an extensive number of health and ageing-related phenotypes. Moreover, these data are available across the 8th decade, a time when risk of dementia and functional decline increases substantially. Critically, the availability of childhood IQ measures enabled us to show that many cross-sectional associations between DNAm PhenoAge and health are confounded by early life cognitive ability.

LBC1936 are a predominantly healthy older ageing cohort, reflected by the young estimation of DNAm PhenoAge compared with chronological age, which might preclude the generalisation of these findings to the broader ageing population. Furthermore, most of the disease assessments within the study are concluded from self-reports, which are often unreliable, limiting their use as indicators of verifiable pathologies. These aspects perhaps hindered additional findings of disease-related associations. In addition, it should be acknowledged that the blood cell profiles and inflammatory markers that DNAm PhenoAge approximates might affect, or be affected by, DNA methylation. Future studies using longitudinal data or causal inference methods may help determine the direction of these associations. Finally, the paucity of data sets with childhood intelligence, later-life phenotypes and methylation data complicates the further replication of our findings.

## Conclusion

We have verified associations between an innovative marker of epigenetic age and a number of pertinent, proxy health-related phenotypes and mortality in older adults. Notably, educational attainment and cognitive ability and age 11 were found to associate with DNAm PhenoAge at age 70. Adjusting models for the latter of these attenuated over half of the late-life associations between health and DNAm PhenoAge by 13–82%. While it does seem DNAm PhenoAge may independently capture some measures of age-related functional fitness and blood-based phenotypes, it does not seem to robustly associate with health phenotypes and is vulnerable to confounding.

## Supplementary information

Supplementary File 1

Supplementary Table 1

Supplementary Table 2

Supplementary Table 3

Supplementary Figure 1

Supplementary Figure 2

Supplementary Figure 3

Supplementary Figure 4

Supplementary Figure 5

Supplementary Figure 6

Supplementary Figure 7

Supplementary Figure 8

## Data Availability

The data set analysed during the current study are available on request from the Lothian Birth Cohort Study, Centre for Cognitive Ageing, and Cognitive Epidemiology, University of Edinburgh. The data are not publicly available as they contain information that could compromise participant consent and confidentiality.

## Code availability

The code used in this analysis (including version information) can be accessed by contacting the corresponding author.
