# Supplementary material for: Childhood intelligence attenuates the association between biological ageing and health outcomes in later life
Source: Transl Psychiatry. 2019 Nov 28;9:323. doi: 10.1038/s41398-019-0657-5 (PMC6883059; doi:10.1038/s41398-019-0657-5)
Supplement: Supplementary file 2 — Supplementary Table 1 [file 41398_2019_657_MOESM2_ESM.docx]

**Supplementary Table 1.** Details of the Lothian Birth Cohort 1936 phenotypes at Wave 1 (~70 years).

**Abbreviations**: SD: standard deviation; WMS: Wechsler Memory Scale; WAIS: Wechsler Adult Intelligence Scale; IPIP: International Personality Item Pool; NEO-FFI: Neuroticism-Extraversion-Openness Five-Factor Inventory; SIMD: Scottish Index of Multiple Deprivation.

| **Phenotype** | **n** | | **%** |
| --- | --- | --- | --- |
| Sex (female) | 440 | | 49.5 |
|  | **n** | **Mean** | **SD** |
| Age (years) | 889 | 69.5 | 0.83 |
| DNAm PhenoAge (years) | 889 | 57.8 | 8.2 |
| **BLOOD** |  |  |  |
| Haemoglobin 130-180 g/L | 885 | 145.6 | 13 |
| Red cell count 4.5-6.5 10^12/L | 884 | 4.62 | 0.42 |
| Haematocrit 0.40-0.54 ratio | 884 | 0.02 | 0.04 |
| Mean cell volume 78.0-98.0 fL | 884 | 91.14 | 4.54 |
| White cell count 4.0-11.0 10^9/L | 884 | 7.11 | 2.34 |
| Neutrophil count 2.0-7.5 10^9/L | 884 | 4.46 | 1.54 |
| Lymphocyte count 1.5-4.0 10^9/L | 884 | 1.91 | 1.44 |
| Monocyte count 0.2-0.8 10^9/L | 884 | 0.53 | 0.19 |
| Eosinophil count 0.04-0.4 10^9/L | 884 | 0.17 | 0.13 |
| Basophil count 0.01-0.1 10^9/L | 883 | 0.04 | 0.03 |
| Platelet count 150-350 10^9/L | 883 | 274.4 | 66.7 |
| Prothrombin time 8.0-11.0 secs | 874 | 10.15 | 2.85 |
| Prothrombin time ratio 0.8-1.2 ratio | 874 | 1 | 0.28 |
| Activated partial thromboplastin time 27.0-38.0 secs | 873 | 28.55 | 3.05 |
| Activated partial thromboplastin time ratio 0.8-1.2 ratio | 874 | 0.94 | 0.12 |
| Fibrinogen 1.5-4.0 g/L | 874 | 3.28 | 0.64 |
| Vitamin B12 200-900 ng/L | 755 | 411.6 | 135.5 |
| Serum folate 5-20 ug/L | 756 | 12.82 | 6.24 |
| Red cell folate 257-800 ug/l | 871 | 413.5 | 126.5 |
| Urea 2.5-6.6 mmol/L | 885 | 6.03 | 1.55 |
| Creatinine 60-120 umol/L | 884 | 78.1 | 17.28 |
| Sodium 135-145 mmol/L | 884 | 140.8 | 2.67 |
| Potassium 3.6-5 mmol/L | 683 | 4.38 | 0.37 |
| Albumin 35-50 g/L | 882 | 44.67 | 3.09 |
| Calcium 2.1-2.6 mmol/L | 880 | 2.35 | 0.09 |
| Triglyceride (0.8 - 2.1 mmol/L) | 803 | 1.63 | 0.76 |
| Cholesterol (mmol/L) | 878 | 5.43 | 1.16 |
| High-density lipoprotein cholesterol 0.9-1.4 mmol/L | 806 | 1.51 | 0.44 |
| Cholesterol: HDLC Ratio | 803 | 3.76 | 1.07 |
| HbA1C 5.0-6.5 %total | 885 | 5.92 | 0.70 |
| C-reactive protein 0-10 mg/L | 879 | 1.22 | 0.87 |
| Thyroid stimulating hormone 0.5-4.7 mU/L | 883 | 2.12 | 1.73 |
| Free thyroxine 9-24 pmol/L | 884 | 15.36 | 2.50 |
| Total triiodothyronine 1.0-2.6 nmol/L | 783 | 2.14 | 0.37 |
| **CARDIOVASCULAR** |  |  |  |
| High blood pressure (yes/no) | 360/529 | - | - |
| High cholesterol (yes/no) | 302/586 | - | - |
| Cardiovascular disease history (yes/no) | 218/671 | - | - |
| Problems with blood circulation (yes/no) | 134/753 | - | - |
| History of stroke (yes/no) | 44/845 | - | - |
| Family history of heart disease, stroke or problems with blood vessels (yes/no) | 547/338 | - | - |
| Mean diastolic blood pressure - sitting | 887 | 81 | 10.26 |
| Mean diastolic blood pressure - standing | 885 | 85 | 10.09 |
| Mean systolic blood pressure - sitting | 887 | 150 | 19.26 |
| Mean systolic blood pressure - standing | 885 | 148 | 19.81 |
| **COGNITIVE** |  |  |  |
| Mini-Mental State Examination (MMSE) total score | 888 | 28.79 | 1.45 |
| WMS III - Logical memory (I + II) total score | 887 | 71 | 18.15 |
| WMS III - Spatial span total | 883 | 14.66 | 2.88 |
| WMS III - Verbal paired associates total score (I + II) | 854 | 26.29 | 9.18 |
| WAIS III - Symbol search | 883 | 24.65 | 6.28 |
| WAIS III - Digit-symbol coding total score | 884 | 56.57 | 12.98 |
| Simple reaction time mean | 885 | 0.28 | 0.056 |
| Log10 transformation simple reaction time mean | 885 | -0.56 | 0.078 |
| Four choice reaction time mean | 884 | 0.64 | 0.085 |
| Inspection time total correct responses | 851 | 112.5 | 10.53 |
| WAIS III - Matrix reasoning total score | 885 | 13.44 | 5.09 |
| Verbal fluency total score | 885 | 42.39 | 12.53 |
| WAIS III - Letter-number sequencing | 879 | 10.89 | 3.21 |
| WAIS III - Digit span backwards | 888 | 7.71 | 2.28 |
| National Adult Reading Test (number correct) | 887 | 34.49 | 8.22 |
| Wechsler Test of Adult Reading (number correct) | 887 | 40.94 | 7.35 |
| WAIS III - Block design total score | 885 | 33.72 | 10.46 |
| **PERSONALITY AND MOOD** |  |  |  |
| Hospital Anxiety and Depression Scale - Anxiety score | 888 | 4.86 | 3.25 |
| Hospital Anxiety and Depression Scale - Depression score | 885 | 2.79 | 2.25 |
| Hospital Anxiety and Depression Scale - Total score | 885 | 7.64 | 4.61 |
| IPIP Extraversion total score | 775 | 21.4 | 7.09 |
| IPIP Agreeableness total score | 774 | 31.04 | 5.41 |
| IPIP Conscientiousness total score | 773 | 28.08 | 6.15 |
| IPIP Emotional stability total score | 772 | 24.57 | 7.70 |
| IPIP Intellect / imagination total score | 771 | 23.89 | 5.79 |
| NEO-FFI Neuroticism total value | 775 | 17.13 | 7.53 |
| NEO-FFI Extraversion total score | 766 | 26.9 | 5.95 |
| NEO-FFI Openness total score | 770 | 25.96 | 5.82 |
| NEO-FFI Agreeableness total score | 775 | 33.42 | 5.29 |
| NEO-FFI Conscientiousness total score | 770 | 34.58 | 6.05 |
| **PHYSICAL** |  |  |  |
| Forced expiratory volume in 1 second | 887 | 2.36 | 0.68 |
| Forced vital capacity | 887 | 3.03 | 0.86 |
| Forced expiratory rate (FEV1/FVC ratio) | 879 | 81.09 | 9.61 |
| Peak expiratory flow | 887 | 351.7 | 133.94 |
| Grip strength (kg) best of 3 in right hand | 885 | 28.98 | 10.19 |
| Grip strength (kg) best of 3 in left hand | 885 | 27.1 | 10.12 |
| Height (cm) | 889 | 166.3 | 8.85 |
| Weight in (kg) | 888 | 76.83 | 14.19 |
| Body Mass Index (kg/m2) | 888 | 27.73 | 4.37 |
| 6 metre walk time in seconds | 885 | 3.86 | 1.22 |
| Demi-span (cm) | 887 | 77.74 | 4.77 |
| Head circumference (cm) | 887 | 57.01 | 2.06 |
| Townsend's Disability Scale score - Activities of daily living | 888 | 1.02 | 2.02 |
| Telomere length | 889 | 4191 | 548 |
| Cancer or tumour (yes/no) | 108/781 | - | - |
| Thyroid disorder (yes/no) | 75/813 | - | - |
| Parkinson's Disease (yes/no) | 5/884 | - | - |
| Arthritis (yes/no) | 395/493 | - | - |
| Allergies (yes/no) | 272/617 | - | - |
| Medical history of gout or allopurinol medication (yes/no) | 31/845 | - | - |
| Diagnosis of diabetes (yes/no) | 70/819 | - | - |
| Leg pain when walking or in bed at night (yes/no) | 345/544 | - | - |
| **LIFESTYLE** |  |  |  |
| Smoking category  -current  -ex  -never | 101  372  416 | - | - |
| Units of alcohol consumer per week | 889 | 10.05 | 13.59 |
| Number of days a month exercise | 778 | 7.66 | 8.09 |
| Level of physical activity  -household chores  -walking etc 1-2 times a week  -walking etc several times a week  -exercise 1-2 times a week  -exercise several times a week  -keep-fit/heavy exercise/sport several times a week | 73  134  396  93  56  25 | - | - |
| Energy in kilo calories daily | 751 | 1913 | 661 |
| SIMD Deprivation Index | 881 | 4648 | 1879 |
| **LIFE-HISTORY** |  |  |  |
| Age-11 IQ (age-11 Moray House Test score corrected for age in days, then converted to IQ) | 838 | 99.82 | 15.27 |
| Number of years of full-time education | 889 | 10.73 | 1.11 |
| Childhood social circumstances | 881 | 0.032 | 2.51 |
| Adult occupational social class   - Professional - Managerial/Technical - Skilled (non-manual) - Skilled (manual) - Partly skilled - Unskilled | 162  321  197  151  34  6 | - | - |
