# Supplementary material for: Childhood intelligence attenuates the association between biological ageing and health outcomes in later life
Source: Transl Psychiatry. 2019 Nov 28;9:323. doi: 10.1038/s41398-019-0657-5 (PMC6883059; doi:10.1038/s41398-019-0657-5)
Supplement: Supplementary file 4 — Supplementary Table 3 [file 41398_2019_657_MOESM4_ESM.docx]

**Supplementary Table 3**. Change in phenotypes over time (standard deviation per year).

SE: standard error

| **Phenotype** | **Standardised β** | **SE** | **p** |
| --- | --- | --- | --- |
| *Grip strength (r)* | -0.035 | 0.002 | <2x10^-16^ |
| *Grip strength (l)* | -0.031 | 0.002 | <2x10^-16^ |
| *Forced expiratory volume (1s)* | -0.059 | 0.002 | <2x10^-16^ |
| *Forced vital capacity* | -0.039 | 0.002 | <2x10^-16^ |
| *Forced expiratory ratio* | -0.033 | 0.004 | <2x10^-16^ |
| *Peak expiratory flow* | -0.053 | 0.003 | <2x10^-16^ |
| *Digit span backwards* | -0.017 | 0.003 | 2x10^-8^ |
| *Symbol search* | -0.042 | 0.003 | <2x10^-16^ |
| *Digit symbol coding* | -0.063 | 0.002 | <2x10^-16^ |
| *Matrix reasoning* | -0.028 | 0.003 | <2x10^-16^ |
| *Letter number sequencing* | -0.044 | 0.003 | <2x10^-16^ |
| *Block design* | -0.044 | 0.003 | <2x10^-16^ |
| *6m walk time (s)* | 0.099 | 0.003 | <2x10^-16^ |
| *Telomere length* | -0.088 | 0.003 | <2x10^-16^ |
