# Supplementary figures and images for: Childhood intelligence attenuates the association between biological ageing and health outcomes in later life
Source: Transl Psychiatry. 2019 Nov 28;9:323. doi: 10.1038/s41398-019-0657-5 (PMC6883059; doi:10.1038/s41398-019-0657-5)

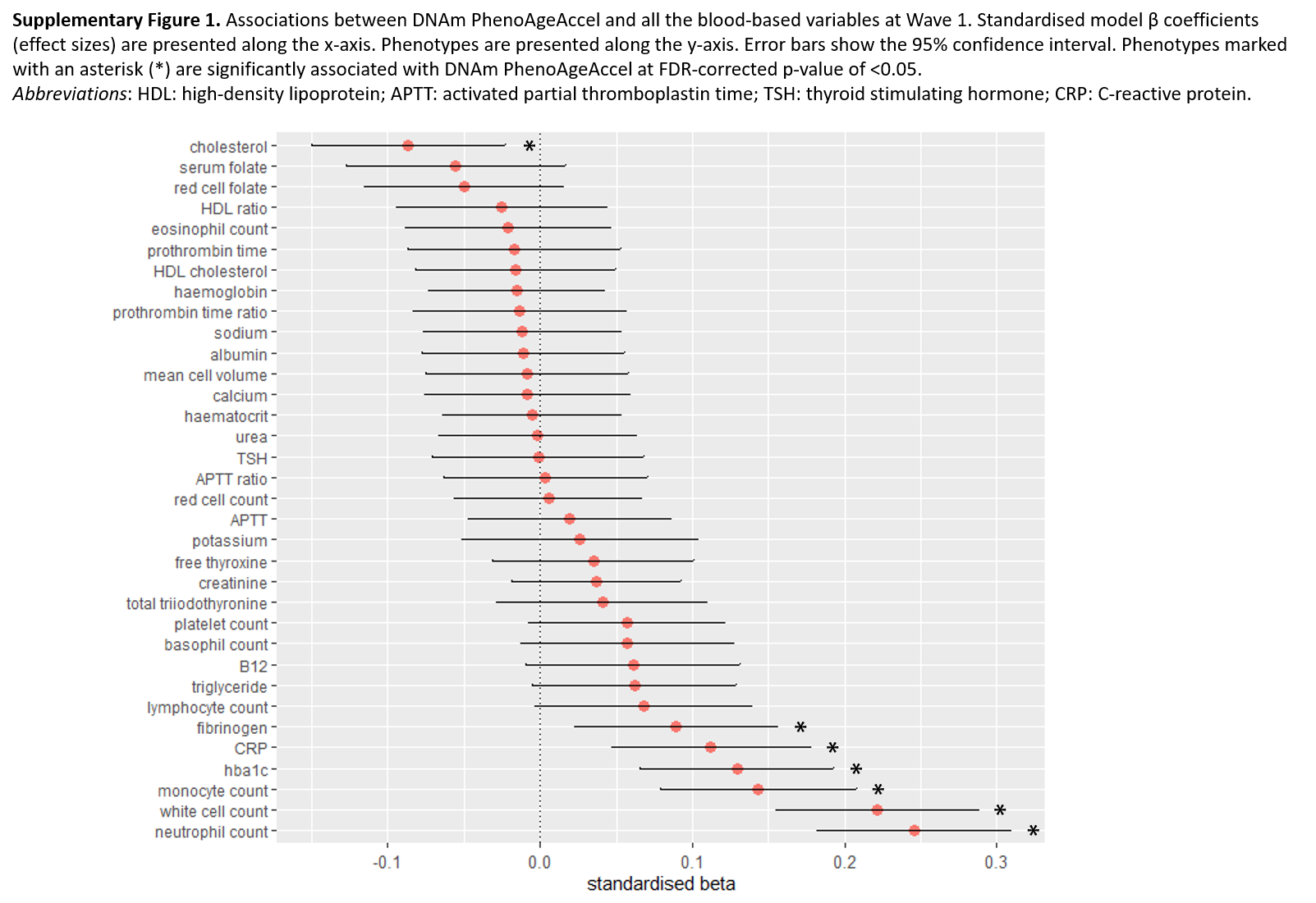

Supplement: Supplementary file 5 — Supplementary Figure 1 [file 41398_2019_657_MOESM5_ESM.tif]

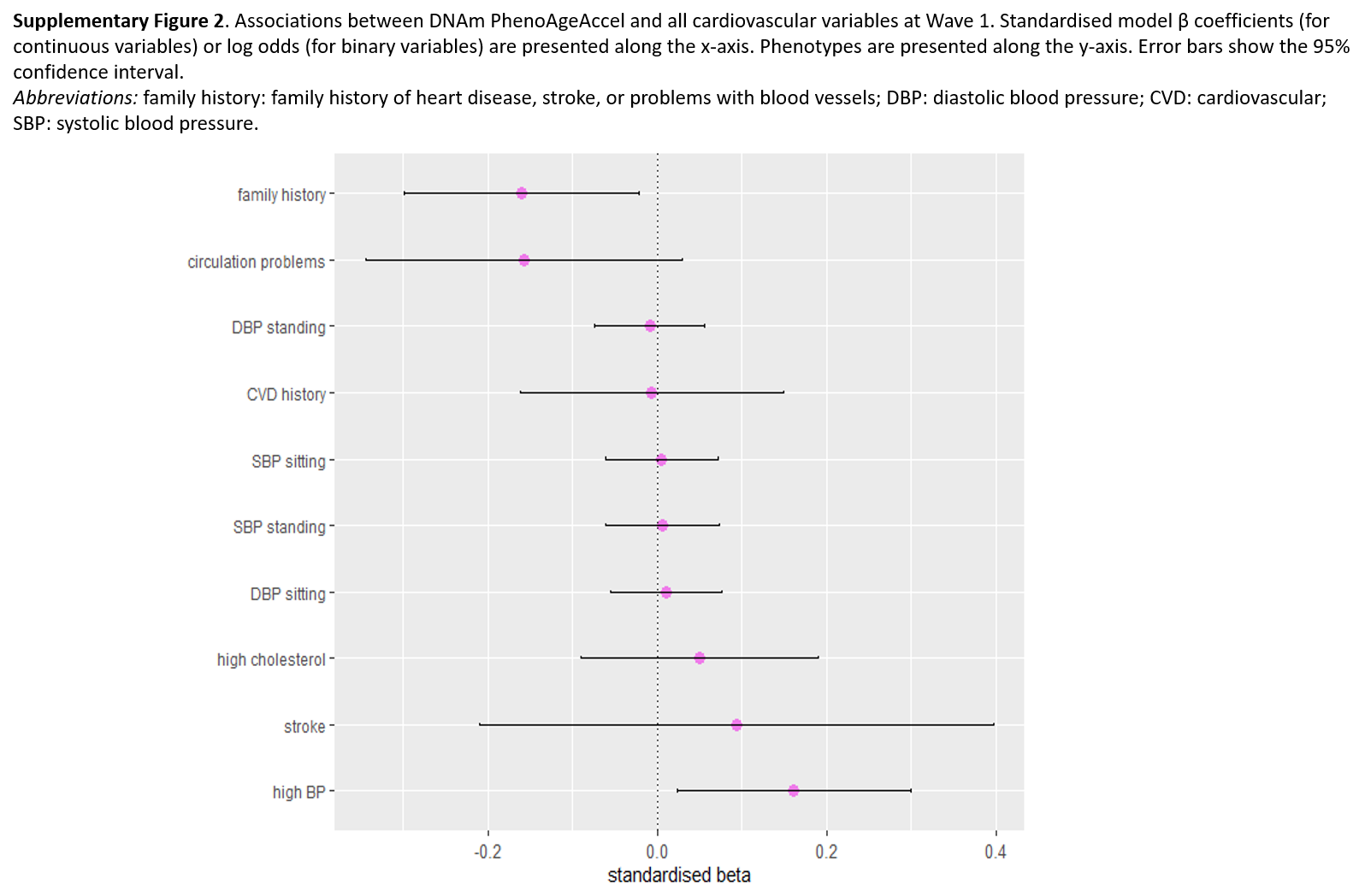

Supplement: Supplementary file 6 — Supplementary Figure 2 [file 41398_2019_657_MOESM6_ESM.tif]

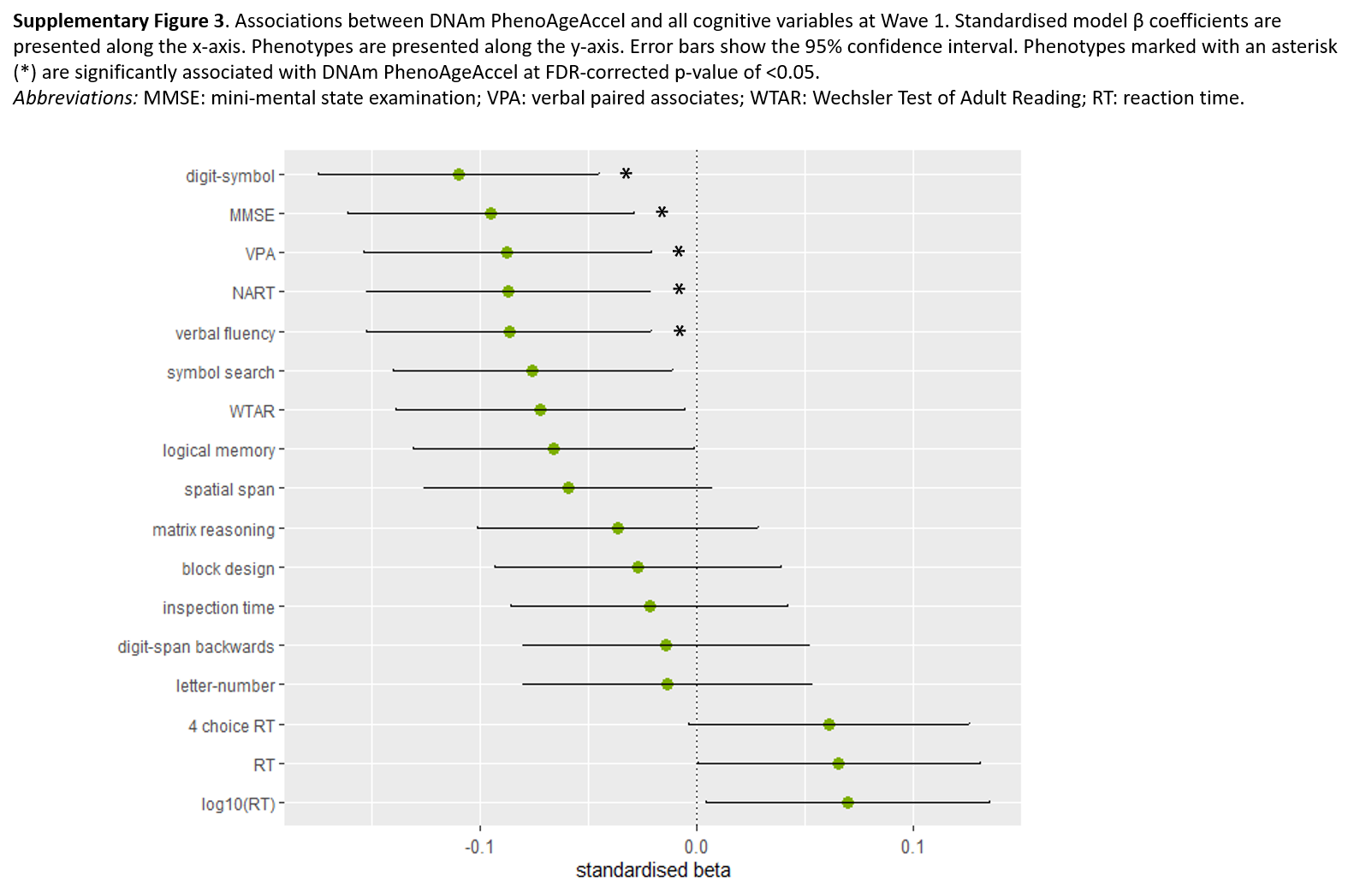

Supplement: Supplementary file 7 — Supplementary Figure 3 [file 41398_2019_657_MOESM7_ESM.tif]

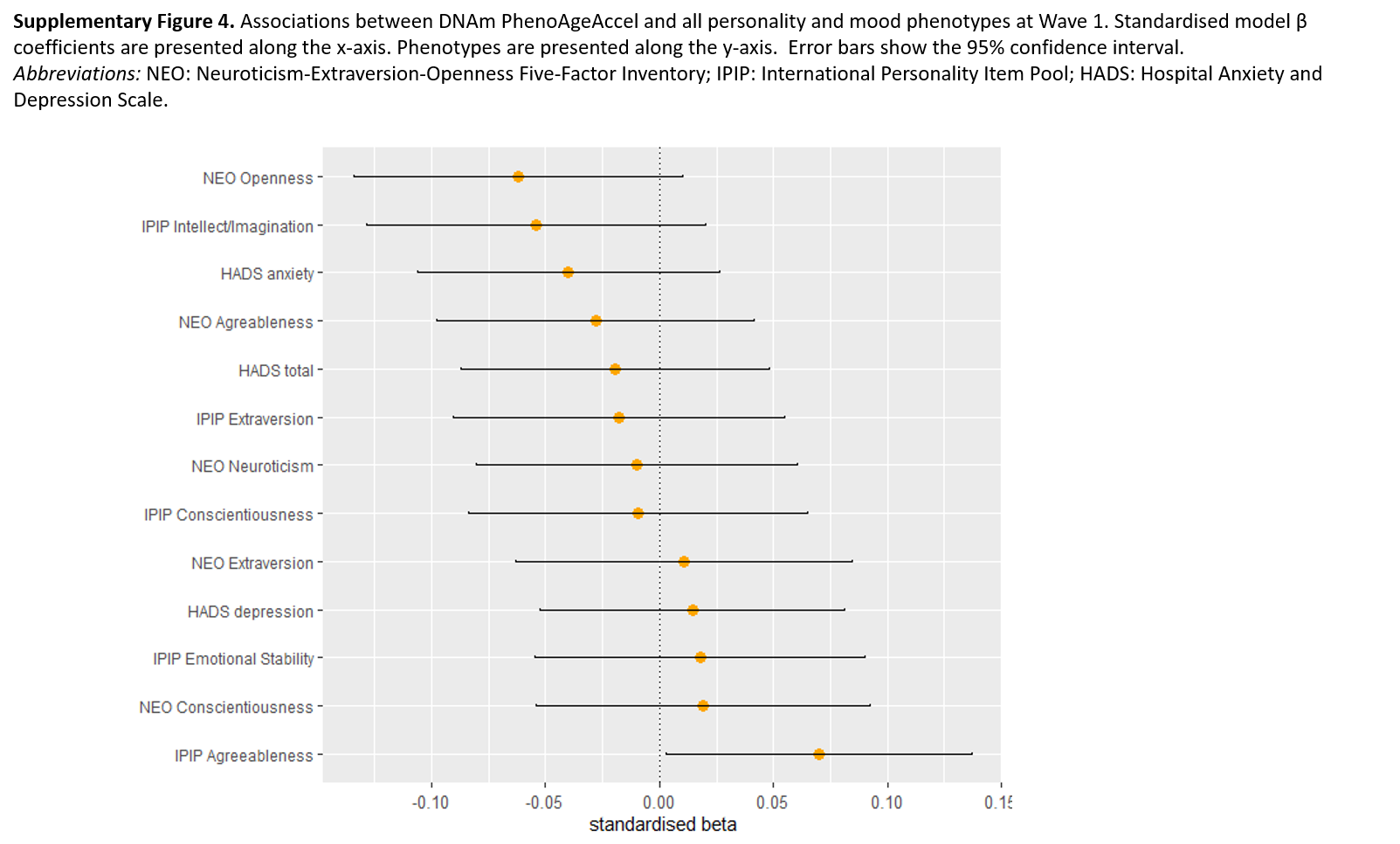

Supplement: Supplementary file 8 — Supplementary Figure 4 [file 41398_2019_657_MOESM8_ESM.tif]

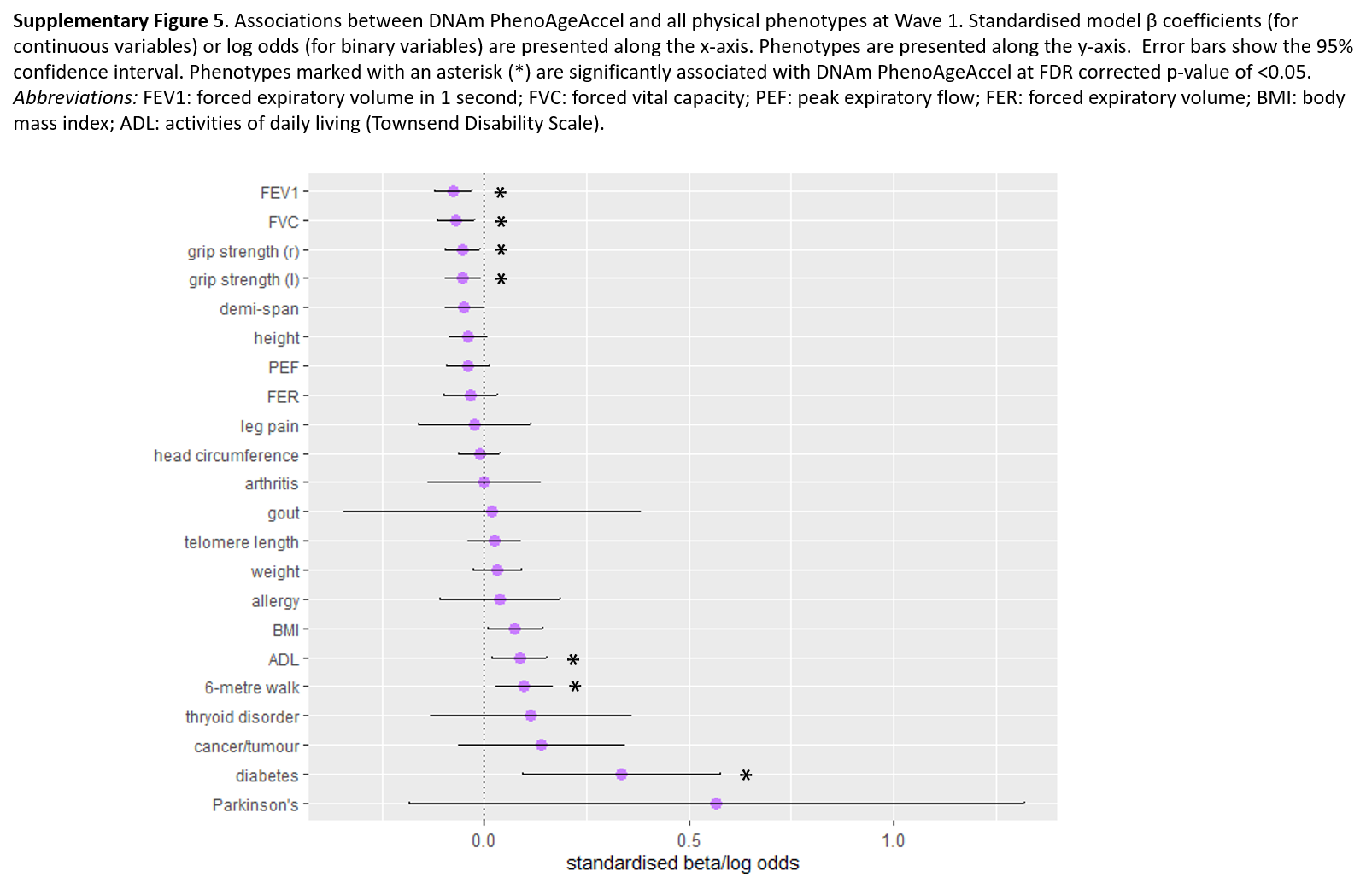

Supplement: Supplementary file 9 — Supplementary Figure 5 [file 41398_2019_657_MOESM9_ESM.tif]

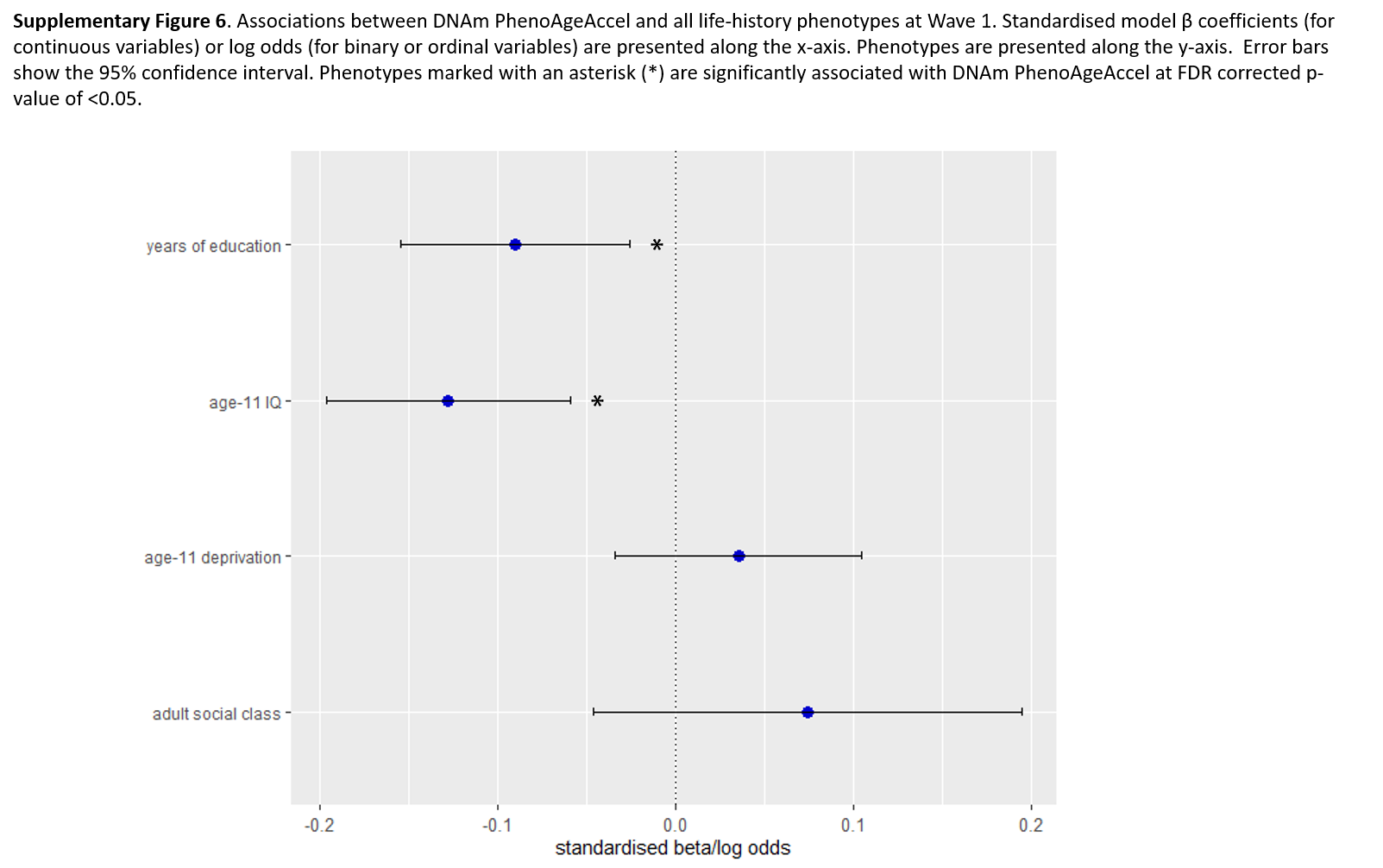

Supplement: Supplementary file 10 — Supplementary Figure 6 [file 41398_2019_657_MOESM10_ESM.tif]

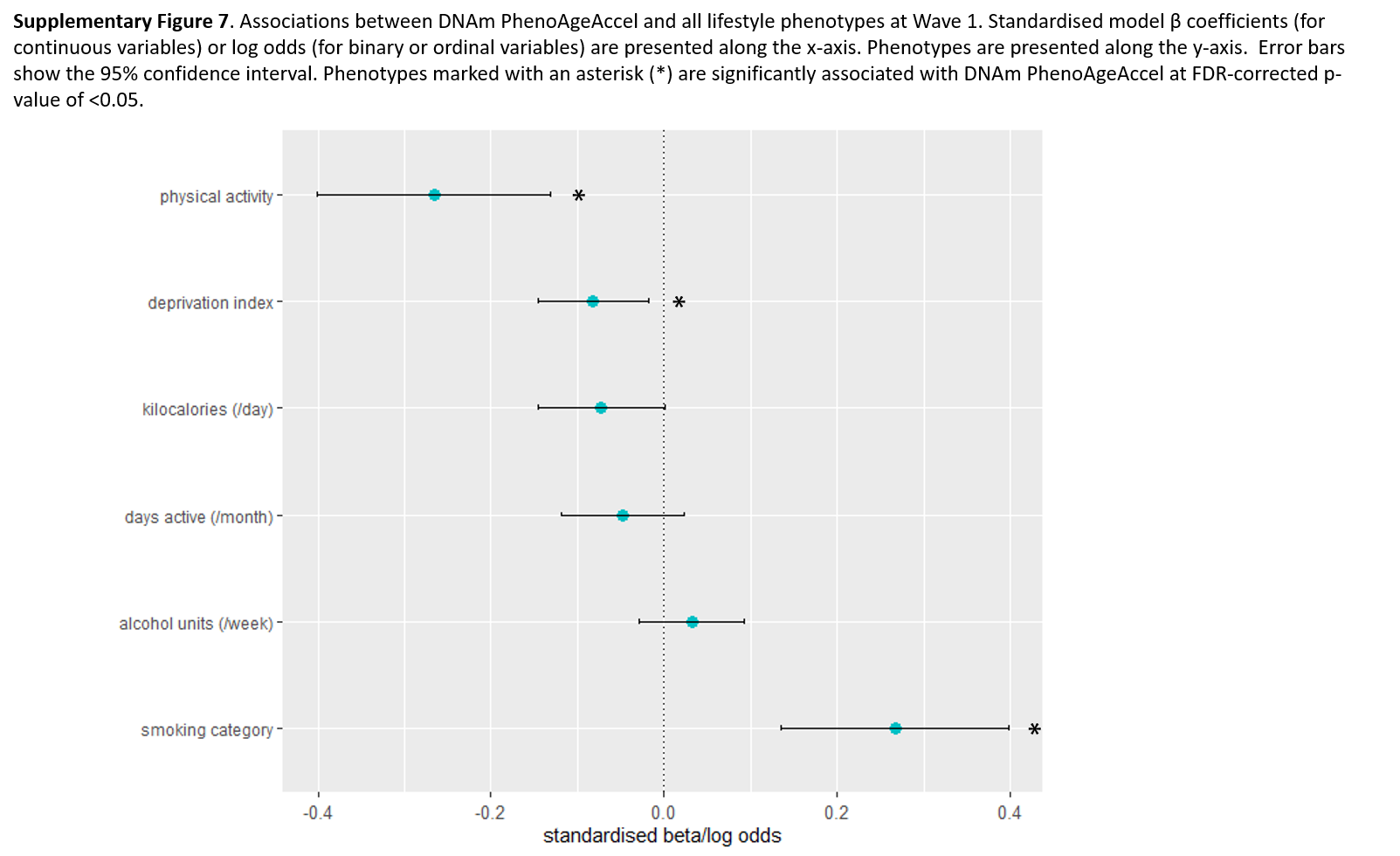

Supplement: Supplementary file 11 — Supplementary Figure 7 [file 41398_2019_657_MOESM11_ESM.tif]

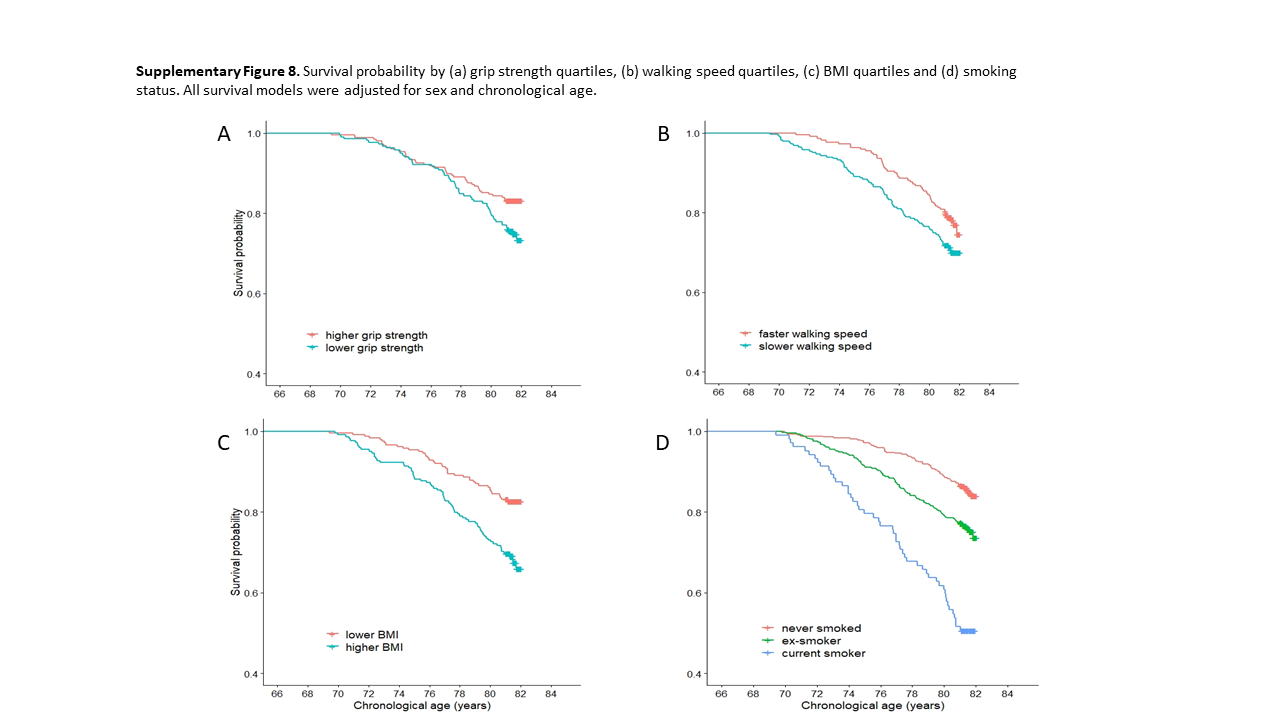

Supplement: Supplementary file 12 — Supplementary Figure 8 [file 41398_2019_657_MOESM12_ESM.tif]
